# Anti-homosexual legislation and HIV-related stigma in African nations: what has been the role of PEPFAR?

**DOI:** 10.1080/16549716.2017.1306391

**Published:** 2017-06-05

**Authors:** Amy Hagopian, Deepa Rao, Aaron Katz, Sallie Sanford, Scott Barnhart

**Affiliations:** ^a^ Department of Health Services, University of Washington School of Public Health; ^b^ School of Law, University of Washington; ^c^ Department of Global Health, University of Washington School of Public Health, Seattle, Washington

**Keywords:** Homonegativity, homophobia, stigma, homosexuality laws, Africa

## Abstract

**Background**: Gay men and other men who have sex with men are disproportionately burdened by HIV infection. Laws that penalize same-sex intercourse contribute to a cycle of stigma, homonegativity and discrimination. In many African nations, laws criminalizing homosexuality may be fueling the epidemic, as they dissuade key populations from seeking treatment and health care providers from offering it.

**Objectives**: We analyzed the ways in which policies and practices of the US President’s Emergency Plan for AIDS Relief (PEPFAR) program addressed pervasively harsh anti-homosexuality laws across Africa. Given the aim of the US PEPFAR program to reduce stigma surrounding HIV, we explored how PEPFAR may have used its influence to reduce the criminalization of homosexuality in the countries where it operated.

**Methods**: We assessed homosexuality laws in 21 African countries where PEPFAR funding sought to reduce the HIV epidemic. We examined PEPFAR Policy Framework agreements associated with those countries, and other PEPFAR documents, for evidence of attempts to reduce stigma by decriminalizing homosexuality.

**Results**: We found 16 of Africa’s 21 PEPFAR-funded countries had laws characterized as harsh in relation to homosexuality. Among the top eight PEPFAR-funded countries in Africa, seven had harsh anti-homosexuality laws. Most (14) of the 16 African ‘Partnership Framework’ (PEPFAR) policy agreements between African governments and the US State Department call for stigma reduction; however, none call for reducing penalties on individuals who engage in homosexual behavior.

**Conclusions**: We conclude that while PEPFAR has acknowledged the negative role of stigma in fueling the HIV epidemic, it has, so far, missed opportunities to explicitly address the role of the criminalization of homosexuality in feeding stigmatizing attitudes. Our analysis suggests mechanisms like PEPFAR Partnership Framework agreements could be ideal vehicles to call for removal of anti-homosexuality legislation.

## Background

Gay men and other men who have sex with men are disproportionately burdened by HIV infection [1,2]. Indeed, while HIV prevalence may be declining in other populations, prevalence is reported to be rising among gay men in many locations, with the risk of infection up to 20 times higher in several African nations. The median HIV adult prevalence in the population of men who have sex with men in Africa, at 15%, is the highest among the World Health Organization’s regions. Most troublingly, gay men and other men who have sex with men often acquire HIV early in life [1].

In many African nations, laws criminalizing homosexuality may be fueling the epidemic, as they dissuade key populations from seeking treatment and health care providers from offering it. High levels of stigma and discrimination are associated with a punitive social and legal environment for men who have sex with men [3–5]. Generalized homonegativity or heterosexism (both words have been used in the literature [6,7]) leads to and is reinforced by criminalization of sexual behaviors. Punitive laws incite deeper levels of stigma. Intersectional stigmas, associated with homosexuality, HIV, poverty and race, interact in several ways to subvert HIV care, prevention and treatment [8,9]. In particular, laws that penalize same-sex intercourse contribute to a cycle of stigma, homonegativity and discrimination, and therefore serve to fuel the epidemic [10]. Men who perceive their behaviors to be associated with shame, judgment, fear or even legal consequences are less likely to disclose sexual behaviors to health care providers, less likely to receive prevention and treatment care, and more likely to contribute to the HIV epidemic [11–13].

In 2012, the independent Global Commission on HIV and the Law, convened by the United Nations Development Program on behalf of the Joint United Nations Program on HIV/AIDS (UNAIDS), produced a report, ‘Risks, Rights and Health,’ which pointed to higher rates of HIV among men who have sex with men in countries where same-sex sexual activity is criminalized, compared to countries where it is not criminalized [14]. Homonegativity is heightened by the physical, psychological or sexual violence against gay men and other men who have sex with men [5,15]. This population suffers extortion, humiliation, discrimination and violence, including rape, based on sexual orientation and gender identity [1,16]. Anti-homosexuality laws interact with homonegativity to reinforce stigma and discrimination, in ways that are counterproductive for HIV prevention, care and treatment efforts.

**Figure 1.**
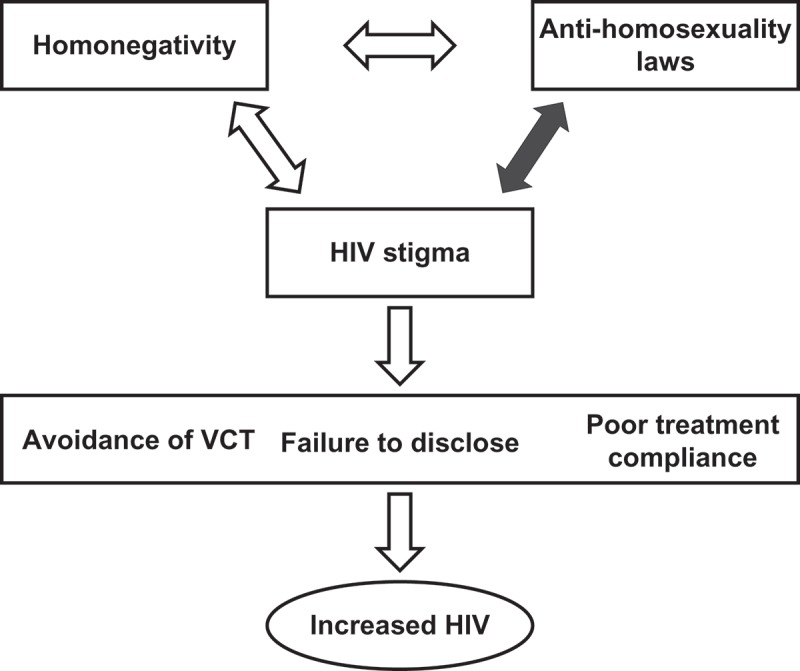
Authors’ conceptual framework of the pathways in which anti-homosexuality laws lead to increased incidence of HIV in a population.

Our conceptual framework of this cycle is illustrated in Figure 1.

Anti-homosexuality laws act to restrict access to services and limit provider efficacy, whether intentionally or not. Some organizations have documented that health care providers have stopped or reduced their scope of services to men who have sex with men owing to fear of harassment [1]. The proportion of gay men and other men who have sex with men who are reached by HIV prevention programs has been in decline in two dozen countries, according to a 2014 report [2]. Another report suggests only 10 percent of this population receives a basic package of HIV prevention interventions [1]. In sub-Saharan Africa, 31 of 45 countries reported no spending on programs that specifically focused on gay men and other men who have sex with men; only 2 of the 45 reported any public domestic spending of that nature [1]. A 2013 analysis found even the US President’s Emergency Plan for AIDS Relief (PEPFAR) has underinvested in prevention programming focused on men who have sex with men relative to the burden of HIV for this group [17]. Although the early activism of gay men pioneered the robust policy response to AIDS in the US and worldwide, homosexual men in sub-Saharan Africa live in politically hostile contexts where their participation and voice are limited [1,2].

PEPFAR founding legislation [18] was promulgated by the George W. Bush administration in 2003 with a $15 billion authorization, and focused on controlling the epidemic through the provision of anti-retroviral drugs in combination with prevention of HIV transmission and care for HIV-positive persons not yet on treatment. The ABC model (Abstain, Be faithful, use Condoms, in order of priority) was noted in the legislation as having prevented HIV transmission in Uganda and other countries. PEPFAR 1.0 emphasized funding relationships with non-government (private) organizational partners, especially faith-based groups [18]. PEPFAR was renewed (and the investment tripled) by the Obama administration in 2008 [19] with a stated emphasis on country ownership and health system strengthening. The first PEPFAR program focused on 15 low-income countries, all but 3 in sub-Saharan Africa, and 8 of them, including Kenya, Nigeria, Uganda, Tanzania, Ethiopia, Zambia, Mozambique and Botswana, are countries that criminalize homosexuality. PEPFAR 3.0 includes 15 African ‘long-term strategy countries’, including Burundi, Cameroon, Cote d’Ivoire, DR Congo, Ethiopia, Haiti, Kenya, Lesotho, Malawi, Mozambique, Rwanda, Swaziland, Tanzania, Uganda, Zambia, and Zimbabwe[20] (see Table 1).

**Table 1.** African PEPFAR countries, their homosexuality laws, their PEPFAR relationships and their HIV rates.

Country	1. 2012 HIV prevalence % (ages 15–49)	2. Authors’ characterization of law’s level of homonegativity*	3. PEPFAR 3.0 long-term strategy country?	4. PEPFAR outlays 2004–2013 (dollars in thousands)	5. Year PF agreement signed	6. Did Partnership Framework address stigma? (NA = no agreement)	7. Did Partnership Framework explicitly address homosexuality criminalization?
Angola	2.3	Harsh	No	84,495	2009	Yes	No
Botswana	23.0	Harsh	No	504,911	2010	Yes	No
Burundi	1.3	Harsh	Yes	34,480	None	NA	NA
Cameroon	4.5	Harsh	Yes	34,731	None	NA	NA
Cote d’Ivoire	3.2	Benign	Yes	623,508	None	NA	NA
D.R Congo	1.1	Unclear	Yes	177,283	2010	Yes	No
Ethiopia	1.3	Harsh	Yes	1,575,485	2010	Yes	No
Ghana	1.4	Harsh	No	90,353	2009	Yes	No
Kenya	6.1	Harsh	Yes	2,582,994	2009/10	Yes	No
Lesotho	23.1	Harsh	Yes	137,324	2009	Yes	No
Malawi	10.8	Harsh	Yes	294,204	2009	No	No
Mozambique	11.1	Harsh	Yes	1,319,325	2010	Yes	No
Namibia	13.3	Unclear	No	570,848	2010	Yes	No
Nigeria	3.1	Harsh	No	2,452,636	2010	Yes	No
Rwanda	2.9	Benign	Yes	726,737	2010	Yes	NA
South Africa	17.9	Protective	No	2,957,043	2010	Yes	NA
Swaziland	26.5	Harsh	Yes	157,564	2009	Yes	No
Tanzania	5.1	Harsh	Yes	1,722,598	2010	No	No
Uganda	7.2	Harsh	Yes	1,814,248	None	NA	NA
Zambia	12.7	Harsh	Yes	1,524,152	2010	Yes	No
Zimbabwe	14.7	Harsh	Yes	311,085	None	NA	NA
Summary	Avg = 9.3%	Harsh = 16	Yes = 15	Avg = 937,904	Signed = 16	Yes = 14; No = 2; no agreement = 5	No = 14 NA = 7

Note: *Based on Law Library of Congress review.

Sources: Column 1: AIDS prevalence data (ages 15–49) come from UNAIDS [21,p. A7–A8]: http://www.unaids.org/sites/default/files/en/media/unaids/contentassets/documents/epidemiology/2013/gr2013/UNAIDS_Global_Report_2013_en.pdf.

Column 2: homosexuality law data come from the Law Library of Congress report, Laws on Homosexuality in African Nations [22], http://www.loc.gov/law/help/criminal-laws-on-homosexuality/african-nations-laws.php.

Column 3: PEPFAR 3.0 document [20].

Column 4: PEPFAR outlays 2004–2013, cumulative, http://www.pepfar.gov/documents/organization/219702.pdf.

Columns 5, 6 and 7: Partnership Framework agreement dates and content, http://www.pepfar.gov/countries/frameworks/index.htm.

PEPFAR 3.0’s strategy document for 2015 includes a ‘human rights action agenda,’ explicitly committing to ‘protecting human rights and addressing the human rights challenges of those affected by the disease.’ PEPFAR provides no guidance as to its views on the criminalization of homosexual activity or the potential role of these criminalization laws in perpetuating HIV stigma or undermining access to care. This is not just theoretical, as 12 of 15 African PEPFAR countries have made it explicitly illegal and punishable with jail time for men to have sex with men. The PEPFAR 3.0 measures of success regarding human rights that are named are relatively weak: (1) expanding access to non-discriminatory HIV care, (2) increasing the role of civil society in advocating for people living with HIV and (3) increasing gender equality.

In 2008 legislation [19] and in 2009 guidance [23], PEPFAR began to emphasize the role of policy reform in PEPFAR partner countries in containing the HIV epidemic. Subsequently, PEPFAR staff negotiated ‘Partnership Framework agreements’ with 22 partner national governments between 2009 and 2012, an accomplishment cited in the PEPFAR 3.0 document [20]. These agreements, while voluntary, were signed at the highest levels of government and were intended to delineate PEPFAR’s five-year financial and technical plans to support HIV prevention, care and treatment programs in partner countries, as well as partner country plans for programming, policy reform and financing [24].

Strategies in these agreements address a range of goals, one of which was reducing stigma [25]. To illustrate, the PEPFAR Partnership Framework guidance document recommends

“Policies address *causes and consequences of HIV-related stigma*” [24] (emphasis added)

and yet no agreements address the decriminalization of homosexuality.

The Global Commission on HIV and the Law has called for a repeal of ‘all laws that criminalize consensual sex between adults of the same sex and/or laws that punish homosexual identity,’ along with establishing other legal protections for men who have sex with men [14]. Analysts have recommended funders use their leverage to support the repeal of laws and policies that criminalize consensual homosexual activity [26,27].

In exploring the legal environment around homosexuality in PEPFAR countries, we hope to inform HIV interventions by highlighting the relationships among homonegativity, anti-homosexuality laws and HIV stigma, and how these work together to fuel the HIV epidemic. The aim of this paper is to examine the ways in which PEPFAR policy and practice were driven by normative assumptions that often go unacknowledged. We asked the question, ‘Given the aim of the US PEPFAR program to reduce stigma surrounding HIV, has PEPFAR leveraged its influence to reduce criminalization of homosexuality in the countries where it operated?’

## Methods

Our methods are grounded in Dunn’s 2015 book, which recommends a ‘methodologically eclectic’ approach to policy analysis, using a ‘reasoned inquiry aimed at finding solutions to practical problems’ [28, p. 3] We began this policy analysis with a review of the literature framing HIV/AIDS stigma in the context of homonegativity, a term referring to negative attitudes towards gay and lesbian people [29]. To inform our conceptual framework, we looked for publications in PubMed that drew the connection between attitudes towards homosexuality and HIV-related stigma (e.g. attitudes towards people living with HIV and AIDS), and how both of these might be associated with the course of the epidemic. We also looked for recommendations from the key HIV/AIDS policy organizations with regard to the role of anti-homosexuality legislation in high-density HIV-positive countries in Africa. We examined both published literature and official reports to find best practice recommendations on policy regarding homosexuality and men who have sex with men in relation to stigma reduction.

Based on this review, we created a conceptual framework (Figure 1). Because the other links in the framework are well established [15,30,31], we elected to focus on the relationship highlighted by the darkened arrow: anti-homosexuality laws and HIV stigma.

PEPFAR teams, comprised of US government staff living in partner countries, negotiated 22 Partnership Framework agreements with 16 African PEPFAR countries [23]. These country-to-country agreements aimed in part to foster a policy environment that would help reduce the pace of the epidemic. Partnership Frameworks were made publicly available at www.pepfar.gov. Our University of Washington (UW) team also had access to the associated PEPFAR country office reporting documents through a cooperative agreement with the U.S. Centers for Disease Control and Prevention (CDC). We analyzed the guidance on what should be in agreements, the agreements themselves and the reporting documents to identify commitments to reduce HIV-associated stigma for all African nations that had agreements.

We then analyzed the 2014 US Law Library of Congress, Global Legal Research Center publication on ‘Laws on Homosexuality in African Nations’ [22], a review of anti-homosexuality laws in African countries, with a focus on the 21 African countries that had received PEPFAR resources. We coded the laws as protective, benign, harsh or unclear. When a law called for jail time for homosexual behaviors (as was the case in most countries in our data-set), we tagged the law as harsh. Only two countries (Cote d’Ivoire and Rwanda) were listed as having ‘no laws against homosexual relations,’ which we labeled as ‘benign.’ Two countries were named by the US Law Library as having laws that were hard to classify, both D.R Congo and Namibia, so we labeled these ‘unclear.’

We created Table 1 to portray, for each of the 21 African PEPFAR recipient countries, the homosexuality law, the various ways in which the country is associated with the PEPFAR program, and the 2012 HIV prevalence rate. For the 16 African countries that signed Partnership Framework agreements, we indicated whether the agreements included language on stigma reduction, and whether that language included any commitments to decriminalize homosexuality as a stigma reduction strategy. Relevant text from both the homosexuality laws and Partnership Frameworks is in Table 2 (supplementary data).

## Results

HIV prevalence rates in the 21 African nations in our study ranged between 1.1% (DR Congo) and 26.5% (Swaziland), with an average of 9.2% among people ages 15 to 49 (see Table 1). HIV prevalence among men who have sex with men is difficult to characterize, as few resources are devoted to measuring it, but the limited studies conducted generally conclude it is significantly higher than in the general population [15]. The average expenditure on PEPFAR programming in the 21 countries between 2004 and 2013 was $940 million per country (median $571 million). Of the 21 PEPFAR countries in our study, 10 had signed Partnership Framework agreements in 2010, 6 had signed agreements in 2009, and 5 have never signed. No agreements have been signed since 2010, although some Partnership Frameworks continue through 2015/16/17 (e.g. Nigeria, South Africa, Haiti).

In 2014, the US Department of State’s Office of the US. Global AIDS Coordinator acknowledged: ‘Human rights among lesbian, gay, bisexual and transgender [LGBT] people in certain parts of the world are increasingly under threat, creating additional barriers to key populations obtaining services’ [20]. Nevertheless, the PEPFAR 3.0 (2014–2018) policy document [20] contains no commitment to supporting civil society and governments of PEPFAR recipient countries to change laws concerning the legality of homosexual status or behavior. The document urges ‘protecting human rights and addressing the human rights challenges of those affected by the disease,’ and commits to ‘end stigma’ and ‘increase access to and uptake of HIV services’ in relation to ‘key populations.’ But the measures of success are quite limited and do not include the very important evidence-based action that partner countries could take to invalidate laws that threaten the human rights of LGBT individuals by criminalizing homosexual acts.

PEPFAR’s policy approach has changed over time. For example, an early PEPFAR strategy through USAID (the Action for West Africa Region, AWARE program) was to promote model omnibus HIV legislation in HIV-affected countries, using ‘best practice’ discourse [32,33]. Between 2005 and 2010, 18 countries in West and Central Africa adopted versions of this model law that included provisions criminalizing transmission of HIV [34], despite evidence such criminalization promotes HIV stigma and other harms [5,35–38]. This model legislation is no longer promoted by PEPFAR, given the preponderance of evidence that criminalization may make HIV epidemics worse.

Laws in 16 African PEPFAR recipient countries are characterized as harsh with relation to LGBT status. Nigeria’s felony conviction for an unnatural offence can trigger a 14-year prison term, and even a public display of ‘same sex amorous relationship’ can garner a 10-year term there. Some Nigerian states have adopted Sharia law that imposes the death penalty for homosexual behavior. Angola and Mozambique prohibit ‘acts against nature’ and punishment in both countries includes ‘disqualification from the practice of a profession.’ In DR Congo, homosexual ‘violations of morality’ are punishable, and in Ethiopia, punishment can be meted out to ‘whoever performs with another person of the same sex a homosexual act, or any other indecent act.’ In Zimbabwe, a male person who, with consent, performs ‘anal sexual intercourse, or any act involving physical contact other than anal sexual intercourse that would be regarded by a reasonable person to be an indecent act’ commits the crime of sodomy. Botswana’s law specifically prohibits anal penetration by a sex organ. Sexual acts between same-sex partners are illegal as ‘sodomy’ violations in Lesotho, Swaziland and Ghana. Ghana also punishes ‘unnatural carnal knowledge.’

In the eastern African nations of Tanzania, Kenya, Uganda, Malawi and Zambia (all former British colonies), laws are similar. In all those countries, it is illegal to have ‘carnal knowledge of any person against the order of nature’ and anyone who ‘permits a male person to have carnal knowledge of him or her against the order of nature’ can be punished. In Zambia, a sodomy conviction can result in 14 years to life in prison. In Tanzania, sodomy convictions can garner 30 years to life imprisonment. Tanzania’s law forbids behaviors that fall short of actual intercourse, and may include masturbation and ‘indecent… behaviour without any physical contact.’

The existence of anti-homosexuality laws does not necessarily mean they are being enforced. However, even with selective and unpredictable enforcement, awareness of these laws and the ever-present threat of enforcement can still fuel stigma, self-stigma (when members of a devalued group internalize stigmatizing beliefs [39,40]) and fear, which feed the arrows in our conceptual framework. A tour of African newspaper websites readily turns up reports of arrests for homosexual activity in PEPFAR-funded countries [41,42]. For example, police recently raided Uganda’s Makerere University, claiming the US-funded Walter Reed HIV research and treatment center was recruiting people into homosexuality [4].

South Africa, Kenya and Nigeria each received between $2.5 and $3 billion between 2004 and 2013, representing the top three PEPFAR-funded countries. Mozambique, Zambia, Ethiopia, Tanzania and Uganda each received between $1 and $2 billion. Among these top eight PEPFAR countries in terms of funding over time, seven have anti-homosexuality laws characterized as ‘harsh,’ while only South Africa’s laws are ‘protective’ [43].

Namibia, which received about $0.6 billion, and DR Congo, with $0.2 billion, had unclear policies. Rwanda, with $0.7 billion in expenditures, and Cote d’Ivoire, with $0.6 billion, were characterized as benign; South Africa was protective. All remaining 16 countries, with a total $15 billion in PEPFAR expenditures from 2004 to 2013, had harsh legal sanctions against men who have sex with men.

None of the 16 Partnership Framework agreements – 7 in 2009, and 9 in 2010 – contained language regarding the decriminalization of homosexuality. Among the signed agreement countries, only one, South Africa, had a ‘protective’ legal climate towards homosexuals or men who have sex with men. Emerging from a long history of racial oppression, South Africa’s post-apartheid Constitution of 1996 took a strong stance against all forms of discrimination including that on the basis of sexual orientation. The South African Constitution declares, ‘The state may not unfairly discriminate directly or indirectly against anyone on one or more grounds, including race, gender, sex, pregnancy, marital status, ethnic or social origin, colour, sexual orientation, age, disability, religion, conscience, belief, culture, language and birth.’ South Africa is a pioneering African nation for protecting people’s rights to equal treatment, including the right not to be discriminated against due to one’s sexual orientation [44–47]. While the South African Constitution protects men who have sex with men, South Africa is still not without its troubles in relation to attitudes towards homosexuality [48].

## Discussion

Widespread homonegativity in Africa has fueled anti-homosexuality legislation, and vice versa. The punitive legal and social environments have served to fuel the HIV epidemic through the mechanisms illustrated in Figure 1. The Global Commission on HIV and the Law called in 2012 for a repeal of all laws that punish homosexual activity or identity [14], ostensibly providing a foundation for global health initiatives to support policy reforms to advance the human rights of gay men and men who have sex with men. International law guards a universal human right to privacy, which protects individuals’ sexual practices from state interference [49]. The Yogyakarta Principles, crafted by the International Commission of Jurists and the International Service for Human Rights at a 2006 meeting in Indonesia, hold that ‘sexual orientation and gender identity are integral to every person’s dignity and humanity and must not be the basis for discrimination or abuse’ [50]. In line with its Zero Discrimination target, U.N.AIDS has similarly called for ‘decriminalizing same-sex sexual practices and ending other punitive laws based on sexual orientation’[1].

There seems to be widespread agreement among global health initiatives that HIV-based discrimination should be prohibited [1,20,24,51,52]. Most international global health organizations have declared that laws, policies, programs and practices should not exclude, stigmatize or discriminate against people living with HIV or their families based on their HIV status. Some, such as PEPFAR, go so far as supporting the implementation of targeted programs for most at-risk population groups, such as gay men and men who have sex with men.

These clear policy goals, however, have not translated into an explicit and effective PEPFAR commitment, or an acknowledgment by partner country governments, that anti-homosexuality laws should be repealed. A 2009 critique of Partnership Framework agreements by Physicians for Human Rights calls out the generally vague language on stigma reduction and the relatively weak guidance on policy changes that might make a significant difference [53]. For example PEPFAR’s Policy Framework guidance document [54] states:

Policies should address causes and consequences of HIV-related stigma, and may support programmatic approaches such as: incorporating Prevention with Positives programs into the training of healthcare workers and lay counselors; utilizing PLWA as lay counselors and peer educators; and employing effective measurement and documentation of stigma in program plans.

That list does not include repealing laws that criminalize homosexuality. Still, even that is progress. Early (2004) PEPFAR funding supported operations by some faith-based organizations that provided anti-gay messaging among their abstinence-only programming [55].

None of the 16 Partnership Framework agreements signed with African countries in 2009 and 2010 contain references to the role of sodomy laws in contributing to stigma and, therefore, advancing the epidemic. None of the provisions of the PEFPAR 3.0 blueprint, even in the human rights agenda section, directly address anti-homosexuality laws.

Supporters of anti-gay legislation in African nations often claim they are resisting the dominant colonialist influence of Western powers. For example, after signing Uganda’s anti-homosexuality act (which was subsequently struck down by the Ugandan Supreme Court for the parliament’s failure to have a quorum at the time of its passage), President Yoweri Museveni referred to gays as ‘disgusting’ human beings, while suggesting that his action was intended ‘to demonstrate Uganda’s independence in the face of Western pressure and provocation’ [56]. Janet Museveni has expressed similar views in her role as a member of parliament [57]. The irony is that while many Western nations are now shedding their anti-gay laws through legislation and judicial pronouncements, scholars trace hostility towards homosexuality and transgender people in Africa to the influence of northern-hemisphere Christian missionaries [58–60], including modern-day missionaries [61,62]. Pre-colonial African cultures were often much more tolerant of sexuality and gender diversity [14]. A recent paper concludes former British colonies (including eight of the PEPFAR 3.0 countries [Kenya, Lesotho, Malawi, Swaziland, Tanzania, Uganda, Zambia, Zimbabwe] [63]) are most likely (compared to countries with other colonial legacies) to have laws that criminalize homosexual conduct [58]. Human Rights Watch has documented that these eight countries directly inherited British Empire laws that criminalize homosexual conduct [64].

There is rich debate about whether it’s appropriate for countries to compel – through aid or otherwise – domestic policy changes in other countries, such as via trade agreements that impose human rights or safety provisions [64]. We acknowledge that Africans may be justly skeptical of policy changes promoted by wealthy countries including former colonial powers [65], and that pressure to change homosexuality laws could be viewed in that light. The LGBT human rights movement, however, has indigenous roots across Africa [66]. In this sense, it is similar to other historical struggles for equality, such as in apartheid South Africa, where in retrospect it’s not hard to know which side was right.

It can be argued the US government used mechanisms other than Partnership Framework agreements to express its support for decriminalization in Africa. Some evidence exists for this, including a suspension of funding to the Ministry of Health in Uganda after President Museveni signed legislation in February 2014 that would have punished gay sex with jail terms up to life. Although a US government spokesperson addressed the media about the suspension of aid [67], we found no documentation of the policy decision on US government websites. Another recent example of extemporaneous opinion on the issue was President Obama’s statements during his 2015 visit to Kenya linking anti-homosexuality discrimination to Jim Crow laws in American history [68].

One exemplary PEPFAR program is the provision of direct support for the Health4Men program in South Africa, which provided services for more than 5000 men who have sex with men and trained almost 1000 health workers in sensitivity and appropriate care [69]. We would argue this program further reinforces our conclusion that decriminalization of homosexuality creates a climate that can reduce HIV prevalence, as South Africa has a uniquely decriminalized legal environment in this context.

Curiously, the 2015 PEPFAR country operational planning reporting guidance [70, p.164] offers a technical code for tracking the activity, ‘Engagement with the government and civil society organizations to reduce criminalization of key populations,’ but otherwise offers no direction to include this activity in work plans. PEPFAR has engaged in programming activity that pushes on culturally sensitive issues, however, as its work on gender equality and gender-based violence has demonstrated [71]. Thus, US-involved bilateral agreements, like the PEPFAR Partnership Framework, which have human resource and infrastructure funds at stake for recipient countries, could be ideal avenues to agree upon anti-discriminatory policies.

Our study has limitations. We relied on a single secondary source of data for information on the legal status of homosexuality. Further, the 2014 report on homosexuality laws in African nations by the US Law Library of Congress noted some nations where harsh laws might be under consideration for change; these included Uganda, which was considering even harsher laws, as well as Angola and Mozambique, where broad criminal code reviews were under consideration. Subsequently, in 2015, Mozambique decriminalized homosexuality [72]. In cases where the law seemed to be in flux, we assumed the law at the time of passage of PEPFAR 3.0 was the applicable statute, since our analysis concerned PEPFAR’s relationship to anti-homosexuality law. We also relied on official reports by PEPFAR, without consideration for behind-the-scenes discussions of policy towards anti-homosexuality laws in PEPFAR recipient countries.

## Conclusions

Widely held negative views about gay men and men who have sex with men in Africa, even among health care workers and academics associated with HIV care and research [73], have restricted optimal support and care for African men who have sex with men, or even research on their behalf [74–77]. Criminalization reinforces those negative views, undermines care and research, and fuels the epidemic. Further, decriminalization is only a mid-term goal in the public health agenda – after that, the focus must turn to eliminating discrimination for gay men in employment, housing and other social determinants of health.

South Africa’s Desmond Tutu has helped Africans understand:

there are no inferior people in his eyes. No one deserves less of God’s love, less of his mercy, or less of his justice…. LGBT people already have God’s full love and acceptance… but they need our acceptance, our love. And to the extent that legal discrimination, those old laws and statutes that make them inferior still exist, it is up to all to work to change those laws. [78]

The US government has condemned African anti-homosexuality legislation in stand-alone statements [79,80], but it has not directly tied such legislation to the effects on the HIV epidemic. We conclude that while PEPFAR has acknowledged the negative role of stigma in fueling the HIV epidemic, it has, so far, missed opportunities to explicitly address the role of criminalization in feeding stigmatizing attitudes. US-negotiated agreements, like the PEPFAR Partnership Framework, could be ideal vehicles to call for removal of anti-homosexuality legislation.

## Paper context

We perused newly available data detailing anti-homosexuality laws in Africa, concerned that criminalization fuels the HIV epidemic. PEPFAR spent nearly $1 billion each in 21 African countries (2004-2013), yet 16 of these countries had harsh anti-homosexuality laws. PEPFAR had high-level policy agreements in 16 countries, and while most condemned stigma, no agreements called for decriminalization. We describe how PEPFAR missed an opportunity to reduce HIV stigma, and therefore slow the epidemic, by promoting decriminalization of homosexuality in Africa.
